# Supplementary figures and images for: Bovine serum albumin detection by using molecularly imprinted surface plasmon resonance sensors
Source: Turk J Chem. 2021 Dec 6;46(2):487–98. doi: 10.3906/kim-2109-6 (PMC10734745; doi:10.3906/kim-2109-6)

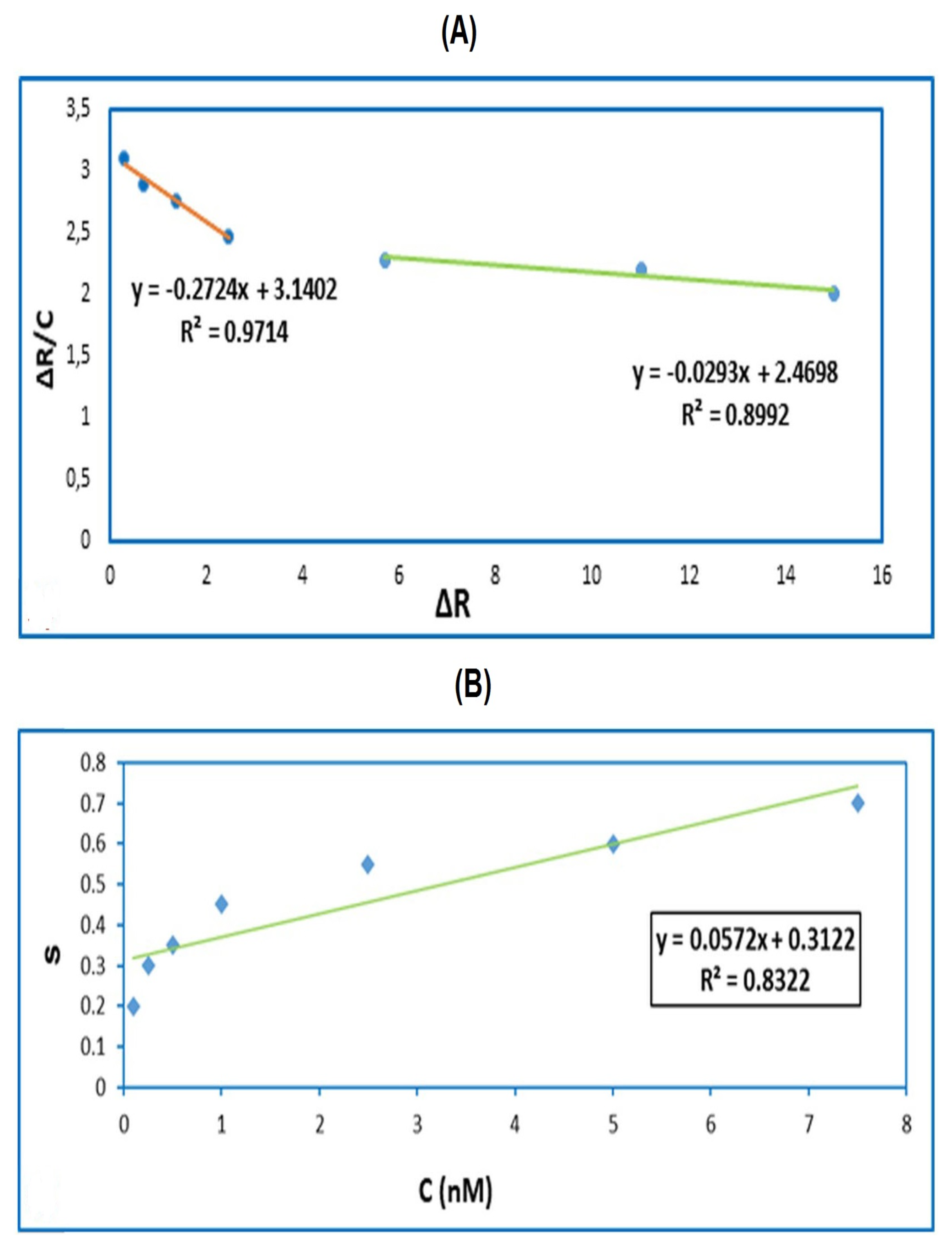

Supplement: Figure S1 — Association kinetic analysis result for BSA imprinted SPR sensors (A) and Determination of kinetic rate constant for BSA with Equilibrium analysis approach (Scatchard) (B). [file turkjchem-46-2-487s1.tif]

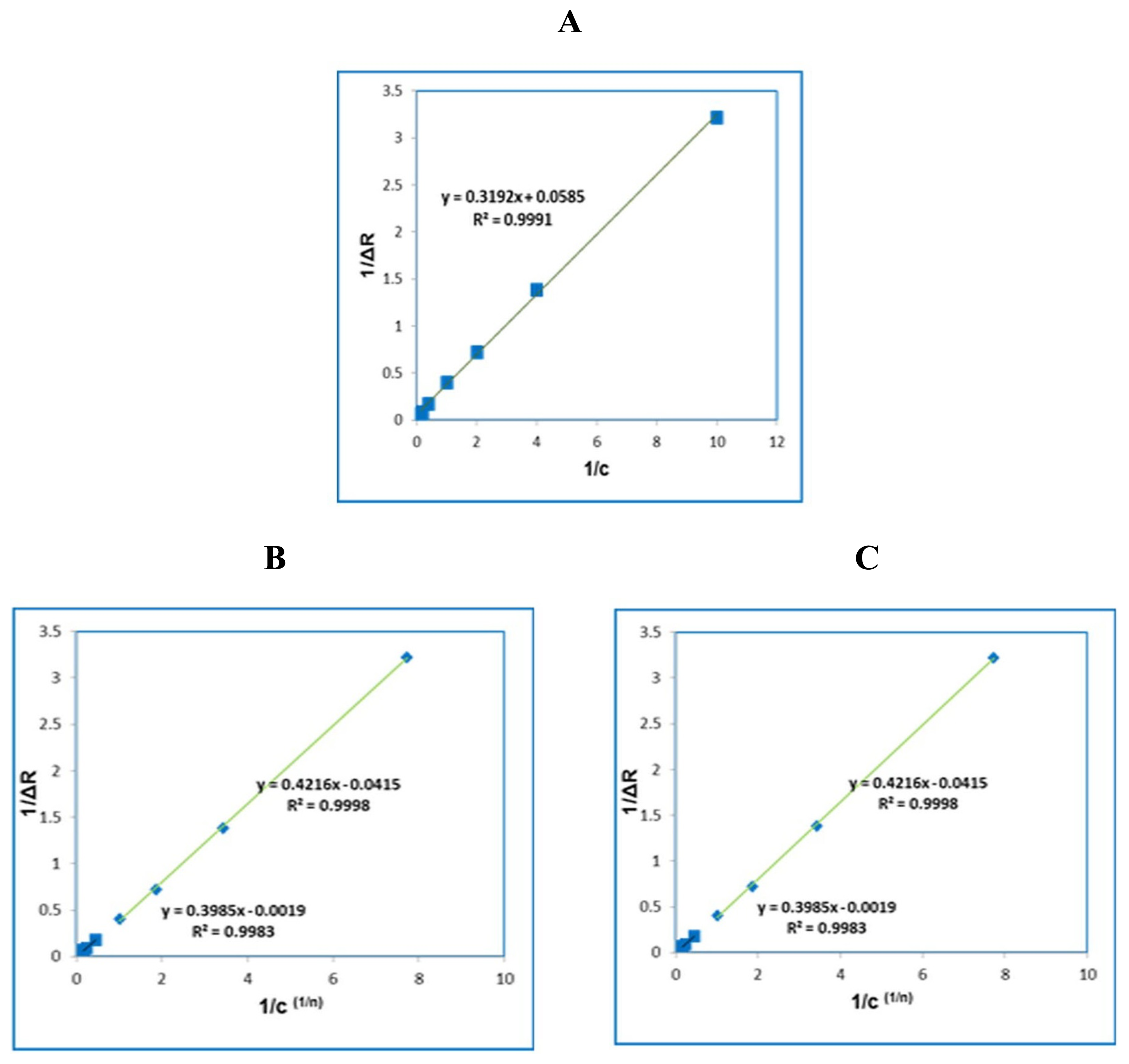

Supplement: Figure S2 — Adsorption isotherm models for BSA adsorption: (A) Langmuir, (B) Freundlich, (C) Langmuir–Freundlich adsorption models. [file turkjchem-46-2-487s2.tif]
